# Trend of leprosy in individuals under the age of 15 in Mato Grosso (Brazil), 2001-2013

**DOI:** 10.1590/S1518-8787.2017051006884

**Published:** 2017-03-29

**Authors:** Bruna Hinnah Borges Martins de Freitas, Denise da Costa Boamorte Cortela, Silvana Margarida Benevides Ferreira

**Affiliations:** I Departamento de Enfermagem. Faculdade de Enfermagem. Universidade Federal de Mato Grosso. Cuiabá, MT, Brasil; II Departamento de Medicina. Faculdade de Medicina. Universidade do Estado de Mato Grosso. Cáceres, MT, Brasil; III Programa de Pós-Graduação. Faculdade de Enfermagem. Universidade de Cuiabá e Universidade Federal de Mato Grosso. Cuiabá, MT, Brasil; Universidade Federal de Mato Grosso, Universidade Federal de Mato Grosso, Cuiabá, MT, Brasil

**Keywords:** Child, Adolescent, Leprosy, epidemiology, Neglected Diseases, prevention & control

## Abstract

**OBJECTIVE:**

To identify the historical trend of leprosy epidemiological indicators in individuals under the age of 15 in the state of Mato Grosso.

**METHODS:**

Descriptive study with trend analysis of leprosy indicators in individuals under the age of fifteen registered in the Mato Grosso’s System for Notifiable Diseases between 2001 and 2013. We used the Prais-Winsten procedure for analyzing generalized linear regression at a significance level of 5%. We considered as increasing time series when the annual percent change was positive, decreasing when negative and stationary when there was no significant difference between its value and zero.

**RESULTS:**

We analyzed 2455 cases of leprosy and the average detection rate in individuals under the age of fifteen was 22.7 per 100 thousand inhabitants. The trend of the general coefficient of incidence was decreasing, with an average annual rate of -5.5% (95%CI -7.5–-3.5). Increasing trend was observed with an increase of 6.7% (95%CI 2.7–10.8) in the proportion of multibacillary cases, 9.4% (95%CI 4.4–14.7) of cases diagnosed with dimorphic clinical form and 14% (95%CI 7.9–20.4) of cases with physical disability level 2 at the time of diagnosis. There was an increasing trend in the average proportion of examined contacts, with a growth of 4.1% (95%CI 1.2–7.1) and average proportion of healing was precarious (39.7%), with stationary trend.

**CONCLUSIONS:**

The historical trend of leprosy cases in individuals under the age of fifteen proved to be decreasing in the period, however the trends of epidemiological indicators such as the proportion of multibacillary cases, physical disability level 2 and healing, indicate late diagnosis with stay sources of transmission and consequent worsening of the disease in the state of Mato Grosso.

## RESUMO

**OBJETIVO:**

Identificar a tendência histórica dos indicadores epidemiológicos de hanseníase em menores de 15 anos no estado de Mato Grosso.

**MÉTODOS:**

Estudo descritivo com análise de tendência dos indicadores de hanseníase em menores de 15 anos registrados no Sistema Nacional de Agravos de Notificação de Mato Grosso, no período de 2001 a 2013. Utilizou-se o procedimento de *Prais-Winsten* para análise de regressão linear generalizada, adotando-se nível de significância de 5%. Consideraram-se como série temporal crescente quando a *annual percent change* era positiva, decrescente quando negativa e estacionária quando não havia diferença significante entre seu valor e o zero.

**RESULTADOS:**

Foram analisados 2.455 casos de hanseníase e o coeficiente médio de incidência em menores de 15 anos foi de 22,7/100 mil habitantes. A tendência geral do coeficiente de incidência foi decrescente, com uma *annual percent change* de -5,5% (IC95% -7,5–3,5). Observou-se tendência de crescimento, com incremento de 6,7% (IC95% 2,7–10,8) na proporção dos casos multibacilares, de 9,4% (IC95% 4,4–14,7) nos casos com forma clínica dimorfa e de 14% (IC95% 7,9–20,4) nos casos com incapacidade física grau 2 no momento do diagnóstico da doença. Verificou-se crescimento na proporção dos contatos de casos novos examinados, com incremento de 4,1% (IC95% 1,2–7,1) e média da proporção de cura considerada precária (39,7%) com tendência estacionária.

**CONCLUSÕES:**

A tendência histórica dos casos de hanseníase em menores de 15 anos mostrou-se decrescente no período. Entretanto, as tendências dos indicadores epidemiológicos, como a proporção de casos multibacilares, de incapacidade física grau 2 e de cura, indicam o diagnóstico tardio com permanência de fontes de transmissibilidade e consequente agravo da endemia no estado de Mato Grosso.

## INTRODUCTION

Leprosy predominantly affects the skin and peripheral nerves. The diagnosis is clinical and epidemiological, performed through anamnesis and dermatologic examination to identify lesions or skin areas with altered sensitivity and/or peripheral nerves impairment (sensory, motor and/or autonomic)^a^. Leprosy diagnosis requires careful examination in children, due to the difficulty of applying and interpreting sensitivity tests^a^.

According to the World Health Organization (WHO), 210,758 new cases of leprosy occurred in the world in 2015. Brazil ranks second in relation to the number of new cases worldwide, with 26,395 records, of which 7.35% were children under 15 years old^26^. Polychemotherapy (PCT) is the main strategy for controlling the disease. Three decades after the treatment started, leprosy cases reduced significantly in Brazil and in the world. The disease remains a national public health problemdespite the improvement in the care of assisted leprosy cases, with a consequent incidence reduction^26^.

In 2015, Brazil had 14.06 cases/100 thousand inhabitants, with 4.28 cases/100 thousand inhabitants in the population under 15 years old. Leprosy has a heterogeneous distribution and is hyperendemic in some states, such as Mato Grosso, which presented 93 new cases/100 thousand inhabitants and 21.3 new cases/100 thousand inhabitants in children under 15 years old also in 2015^14,b^.

In the last decades, the incidence of registered cases in children maintained or discreetly reduced. An Indian study found that the leprosy incidence in children under 15 years old showed an insignificant decrease from 2003 to 2012, with more cases between two and 15 years old^21^. However, other countries indicated a reduction in leprosy cases in this age group. China (1987-2008) decreased from 186 to 40 cases in absolute number, and Zambia (1991-2009), from 27.3 to 4.3/100 thousand inhabitants^11,25^. A Brazilian study (Fortaleza, CE) showed general average growth trend over the course of 13 years (1995-2007), with an average of 95 cases per year^1^.

The strength of morbidity, magnitude and trend of the endemic of the children population is considered the main indicator of the disease monitoring, since it suggests intense circulation of *Mycobacterium leprae*, active and recent transmission, besides index cases not yet identified and not assisted by the health system^8,26^.

The contact of individuals with leprosy in the bacilliferous form is the main source of disease transmission, mainly in the household environment. In endemic countries, the child population generally has early contact with a bacilliferous patient^20^. As the risk of a healthy subject developing leprosy increases ninefold when a family member is affected, performing the household contact examination of all newly diagnosed cases^23^ is recommended. Collective examination is another essential strategy for controlling the disease. One study added 38.2% of cases to spontaneous demand through active school search, indicating that this environment is an institutional area for active leprosy control actions in this population^5^.

Epidemiological indicators, such as the leprosy incidence coefficient in children under 15 years old, the proportion of cure of new cases and the proportion of intradomiciliary contacts of new examined cases, allow us to monitor the extent of disease elimination as a public health problem. In addition, these indicators support the critical analysis of the results and help in the decision-making process, contribute to the continuous improvement of organizational processes, and enable the comparative analysis of performance^a,c^.

Studying leprosy indicators in children under 15 years is done to understand the magnitude and strength of endemic and health system performance in disease surveillance. Data organization in a time series allows us to analyze the phenomenon, possibly indicating evolution of risks to which people are or have been subjected, providing subsidies for causal explanations, assisting in health planning and evaluating the impact of interventions^17^.

As such, this study aimed to identify the historical trend of leprosy epidemiological indicators in individuals under the age of 15 in the state of Mato Grosso.

## METHODS

In this descriptive study, we analyzed the historical trend of leprosy indicators in individuals under the age of 15 reported in the state of Mato Grosso, from 2001 to 2013.

The historical series is a sequence of quantitative data relating to specific moments and studied according to their distribution in time. It had the purpose of detecting and interpreting the evolution of leprosy indicators during this period^17^.

We obtained information on children less than 15 years with leprosy through a database of the National Notification System of Mato Grosso (SINAN/MT), provided by the epidemiological surveillance department of the State Health Department of Mato Grosso (SES/MT, 2014). To select the historical series, we included all the years (2001-2013) available in the SINAN/MT bank until the collection date. A historical series should refer to a period sufficient to enable the researcher to perceive the event trend: whether stationary, ascending or descending. However, the period is generally determined by the data availability, not by the researcher^17^.

The historical series analysis included leprosy cases that presented one or more of the following cardinal signs: a) lesion(s) or skin area(s) with altered sensitivity; b) impairment of peripheral nerve(s), with or without thickening, associated with sensory, motor or autonomic alterations; and c) positive intradermal smear microscopy^a^. We excluded the cases considered diagnostic error in the database, with transfer to another state or country, duplicity and data inconsistency. Those excluded due to data inconsistency were the cases with birth date corresponding to the diagnosis date, making up the zero year of age.

The variables of interest were the indicators that represent the strength of morbidity and magnitude of leprosy (incidence coefficient in children under 15 years) and the quality of actions and services (proportion of healing of new cases and proportion of new contacts of cases). The other variables were temporal characters (years), sex, operational classification, clinical form, physical disability at diagnosis and way of detection.

Leprosy incidence coefficient in the population was estimated (per 100 thousand inhabitants) and calculated according to the relative population of the 2000 and 2010 censuses, in relation to the age group, according to the demographic data of the Brazilian Institute of Geography and Statistics (IBGE, in Portuguese), counting in 1996 and with intercensal projections^b^. The incidence coefficients among those younger than 15 years old were classified as: hyperendemic (≥ 10.00); very high (9.99 to 5.00); high (4.99 to 2.50); mean (2.49 to 0.50); and low (< 0.50). The assessments of the proportion of healing and proportion of intradomiciliary contacts of new cases occurred from 2003 to 2013 due to the need of following-up the cohort according to calculation recommended by the Ministry of Health^c^. The reference parameters for the healing ratio were good (≥ 90%); regular (89.9% to 75%); and precarious (< 75%). The reference parameters for the proportion of intra-household contacts of new leprosy cases were good (≥ 75%); regular (74.9% to 50%); and precarious (< 50%)^a^.

To characterize temporal trends, we transformed the logarithmic coefficients (Y), as it facilitates to reduce the heterogeneity of the residual variance of the linear regression analysis, that is, the values of the difference between the points of the mean line and points of the time series. In addition, this transformation contributes to determine the annual growth rate. We used the *Prais-Winsten* procedure for generalized linear regression analysis, since it allows us to estimate the regression coefficients with correction of the first-order temporal autocorrelation^2^.

The regression equation of the time series associates the dependent variable with the year. Thus, for each “I” year included in the study period, there is *logY*(*i*) = *a* + *bi* and *logY*(*i* + 1) = *a* + *b*(*i* + 1). The “*a*” value corresponds to the intersection between the line and the vertical axis and the “*b*” value correspond to the slope of the line, which was estimated by linear regression analysis. Therefore, by difference: *logY*(*i* + 1) - *logY*(*i*) = *b*(*i* + 1 - *i*) = *b*. Thus, we calculated the value of the coefficient “*b*” and the standard error “*EP*” of the regression analysis and calculated the annual percent change (APC) and confidence interval (95%CI) using the APC = -1 + 10^*b* and 95%CI of that rate = -1 + 10^(*b* ± *t* * *EP*), where “*t*” is the tabulated value of the Student t distribution. When the rate was positive, the time series was considered increasing; when negative, it was considered decreasing; when there was no significant difference between its value and zero, it was stationary^2^. The software Stata 11.1 performed the statistical analysis.

The Research Ethics Committee of the State Health Secretariat of Mato Grosso approved this study, under Opinion 491,444, following all the provisions of Resolution 466/12.

## RESULTS

From 2001 to 2013, SINAN/MT reported and recorded 2,567 new leprosy cases in children under 15 years old. Of these, we excluded cases with a diagnosis error (n = 65), with a transfer to another state or country (n = 12) and data inconsistency (n = 16), totaling 2,455 registered cases of leprosy in children less than 15 years old in Mato Grosso, with a mean of 188.8 cases per year. The mean coefficient of disease incidence in this age group was 22.7/100 thousand inhabitants in the period.

**Figure 1:**
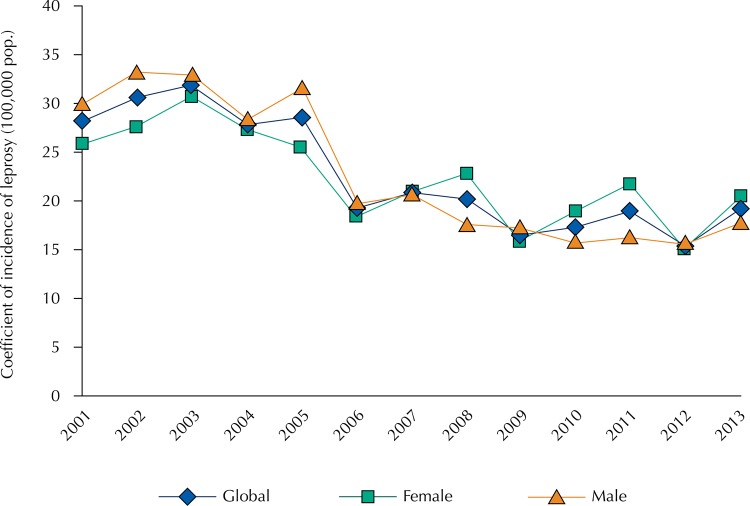
Time series of the coefficient of incidence of leprosy in children under the age of 15 in relation to sex. Mato Grosso, Brazil, 2001-2013.

A decrease in the general trend of the disease incidence coefficient occurred, with an APC of -5.5% (95%CI -7.5–-3.5). Males had more pronounced decrease, with an APC of -6.5% (95%CI -8.9–-4.1). However, the overall decrease occurred from 2005 to 2009 (Figure 1).

The Table shows the trend and the APC of leprosy cases in children under 15 years old according to operational classification, clinical form, physical disability and way of detection. We observed increasing trend in cases with multibacillary operational classification, dimorphic clinical form (D), detection by contact examination and level 2 physical disability at the time of diagnosis. However, the trends for those with absence of physical disability and level 1 physical disability at the time of diagnosis remained stationary (p > 0.05).

**Table:** Trend and annual percent change (APC) in leprosy cases in children under 15 years old in relation to operational classification, clinical form, physical disability and detection. Mato Grosso, Brazil, 2001-2013.

Variable	APC (%)	95%CI	Trend
Operational classification			
Paucibacillary	-4.3	-7.1– -1.5	Decreasing
Multibacillary	6.7	2.7–10.8	Increasing
Clinical form			
Indeterminate	-3.8	-6.2– -1.3	Decreasing
Tuberculous	-3.9	-7.3– -0.3	Decreasing
Dimorphic	9.4	4.4–14.7	Increasing
Lepromatous	-6.7	-10.6– -2.7	Decreasing
Ignored^a^	16.0	4.1–29.2	Increasing
Physical disability			
Level Zero	-0.2	0.5–0.8	Stationary
Level 1	4.2	-1.4–10.1	Stationary
Level 2	14.0	7.9–20.4	Increasing
Ignored^b^	-3.8	-7.3– -0.2	Decreasing
Detection mode			
Forwarding	-3.3	-6.1– -0.3	Decreasing
Spontaneous demand	-1.1	-3.7–1.5	Stationary
Collective Examination	-2.9	-7.7–2.1	Stationary
Contacts examination	6.3	3.9–8.7	Increasing
Other forms	1.7	-4.8–8.7	Stationary
Ignored^a^	1.0	-6.6–9.1	Stationary

^a^ Ignored or unfilled field.

^b^ Ignored, unfilled and unapplied assessment field.

**Figure 2:**
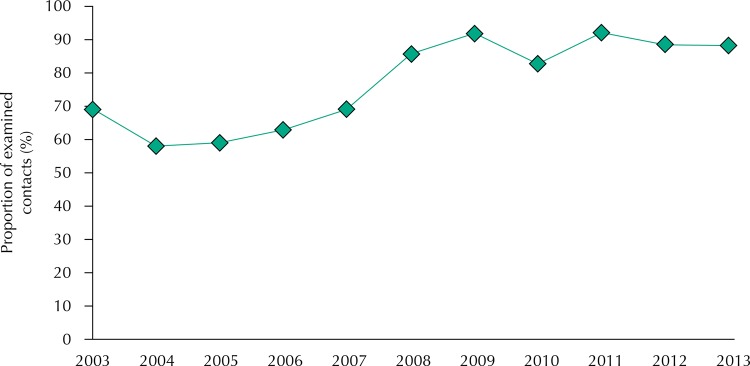
Time series of the proportion of examined contacts. Mato Grosso, Brazil, 2003-2013.

**Figure 3:**
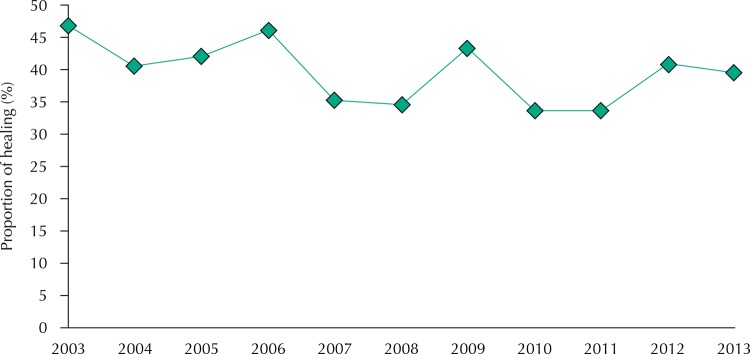
Time series of the proportion of healing of leprosy cases in children under 15 years old. Mato Grosso, Brazil, 2003-2013.

Figures 2 and 3 show, respectively, analyzes regarding the ratio trend of examined contacts and the proportion of healing treatment cases. The proportion of examined, recorded contacts showed an increasing trend, with APC of 4.1% (95%CI 1.2–7.1) in the period. The healing ratio indicator of the new cases showed a stationary trend, with APC of -1.7% (95%CI -3.8–0.4). The mean proportion of contacts examined in the period was 77.1% and the healing proportion was 39.7%.

## DISCUSSION

Among the national leprosy control strategies, defining and monitoring of areas with a higher risk of disease detection are necessary. Although the incidence coefficient trend in children under 15 years old decreased in the studied period in the state of Mato Grosso, it remained hyperendemic, with an increase in multibacillary cases, especially in the dimorphic clinical form. The cases with level 2 physical disability at the time of diagnosis increased, and it was precarious in relation to the healing rate of new cases, with a stationary trend during the analyzed years. These results indicate the recent endemic transmission and deficiency in the care and follow-up of new diagnosed cases until the completeness of treatment.

A study conducted from 1996 to 2007, in Mato Grosso, also described hyperendemicity in children under 15 years old, with a slight decrease in cases^19^. For every 100 thousand inhabitants, there were 33.3 cases in 1996-1999, 33.2 in 2000-2003 and 26.7 in 2004-2007^19^. In those years, leprosy hyperendemicity in children under 15 years old indicates bacillus persistence and difficulty of the programs to control the disease. Leprosy is potentially disabling when it affects childhood because of the possibility of deformities; this is a period of growth and development, and can influence school life due to social limitation, discrimination, low self-esteem and stigma. These individuals experience complex feelings and situations in daily life, their routine changes by the limits dictated by the disease and treatment^18,24^.

Research in other countries has shown recent decrease in the disease trend in this population^11,13,26^, as in this study. However, from 1980 to 2003 the state of Espírito Santo showed a significant increasing trend for the coefficients in children under 15 years old (increase of 7% per year)^15^.

In Mato Grosso, the decreasing trend in the leprosy incidence may be due to the supply of PCT treatment in the health units. Economic growth, improvements in social areas and reversal of the care model for the family health strategy in the last decades may have contributed to the reduction of the local endemic disease^9^.

This decreasing trend was not progressive in the period, concentrating between 2005 and 2009. This probably occurred due to the actions developed by the “Projeto Prioritário Tolerância Zero: Mato Grosso sem hanseníase” (Zero Tolerance Priority Project: Mato Grosso without leprosy) (SES/MT/2001), launched in 2001 with the aim of accelerating activities to achieve the goals of eliminating the disease through financial incentives. This process initially causes an increase in detection for its active search, but it tends to fall due to the reduction in transmissibility resulting from the treatment and healing of the cases^22^.

In that period, the correction in the calculation of the incidence coefficient by the National Leprosy Control Program (PNCH) also occurred, including as new cases only those present in the SINAN database, at federal level, until January 15 of the following year, instead of March 31, as done previously. This operational change favored the modification in the structure of the historical series, producing an artificial fall in the number of new cases^d^.

Some studies suggest that the increase in cases is due to operational improvements in disease control, which increases the diagnoses^6,19^. In the period, control activities were in effect in the state due to the policy of elimination of the disease, and generated a 105.7% increase in units for leprosy diagnosis and treatment^e^.

The historical series of multibacillary cases annually increased in 6.7%. The decreasing trend of paucibacillary cases and increasing multibacillary cases suggests late diagnosis. The paucibacillary cases represent initial leprosy phase, which is desirable to conclude the diagnosis, since the bacillary load is low and lack to act as a source of infection, being relevant for endemic control. However, the identification of multibacillary cases, characterized as contagious due to the high bacillary load and the high risk of transmissibility, suggests health services failure^10^.

The increasing trend of cases defined as ignored as to the clinical form suggests impairment of diagnosis or incompleteness of the data due to lack of preparation or lack of professional commitment. Other studies also observed an increase in the number of fields ignored in the Brazilian epidemiological surveillance system, not identifying significant advances regarding completeness, which generates deficient and unreliable data, as well as interfere in the knowledge of the health-disease process^3,4^. Completeness is a guarantee of quality information, an ideal condition for the objective analysis of the event under study.

The increasing trend of physical disability level 2 at the time of leprosy diagnosis reflects the disease evolution time, the low effectiveness of early detection and the hidden prevalence^12^. Physical disability level 2 indicates the presence of visible lesion and suggests underreporting; it is used to assess the delay of diagnosis and signals to deficiency of health services in the early and accurate detection of cases in children under 15 years old, which may negatively influence social interaction through the increase of stigma and prejudice^18^.

The improved global strategy for additional reduction of the 2011-2015 leprosy burden emphasized the importance of implementing disease control actions and strengthening training programs for health professionals. Therefore, the improvement of diagnostic activities and the follow-up of leprosy cases, especially in children under 15 years old, favor the reduction of disabilities and inconsistencies in the health information system of the disease^16,26^.

As new cases in children may indicate those undiagnosed and not assisted by health units, the trend growth of detecting cases with household contact examination is relevant for the early diagnosis, contributing to the reduction in the transmission of Hansen’s bacillus.

The average proportion of contacts of these new cases was considered good according to the reference standard. The increasing proportion trend suggests an improvement in epidemiological surveillance activities. On the other hand, the trend of healing proportion was stationary in the period, with a precarious average, possibly resulting from unprepared health services to ensure adherence to treatment until medical discharge or poor quality of follow-up of cases recorded in the SINAN/MT^a^, or both.

We consider the possibility of underreporting of cases, which is due to problems in the flow of receiving information, possibility of errors during data transfer, and lack of completion of investigation and notification forms^7^. This would further increase the size of the disease in the state.

The study has other limitation, such as the impossibility of correcting typing errors. In addition, some data in the SINAN/MT may be underestimated, as well as the information of the patient’s monitoring by the health units, which would explain the low average of healing rate.

We concluded that in Mato Grosso the leprosy incidence trend in children less than 15 years old in the studied period decreased, but the average detection rate of cases in children less than 15 years remains hyperendemic, with a trend to increase multibacillary cases, the dimorphic clinical form and with physical disability level 2 to the diagnosis. There are conditions for continuous transmissibility of active cases and late detection, and the healing rate is lower the desired, which may culminate in larger transmission outbreaks and the onset of complications such as disabilities and physical deformities.

## Footnotes

a Ministério da Saúde (BR). Secretaria de Vigilância em Saúde. Departamento de Vigilância das Doenças Transmissíveis. Diretrizes para vigilância, atenção e eliminação da hanseníase como problema de saúde pública: manual técnico operacional. Brasília: Ministério da Saúde, 2016.

b Ministério da Saúde (BR). Sala de Apoio à Gestão Estratégica (SAGE).Situação epidemiológica da hanseníase no Brasil (SINAN/SVS/MS) [cited 2017 Jan 11]. Available from: http://sage.saude.gov.br/

c Ministério da Saúde (BR), Secretaria de Gestão Estratégica e Participativa, Departamento de Articulação Interfederativa. Caderno de diretrizes, objetivos, metas e indicadores: 2013-2015. 2 ed. Brasília (DF); 2014.

d Ministério da Saúde (BR), Secretaria de Vigilância em Saúde, Departamento de Vigilância Epidemiológica. Programa Nacional de Controle da Hanseníase. Brasília (DF); 2007. (Nota técnica nº 010/2007/PNCH/DEVEP/SVS/MS).

e Ministério da Saúde (BR), Secretaria de Vigilância em Saúde. Sistema nacional de vigilância em saúde: relatório de situação: Mato Grosso. 2. ed. Brasília (DF); 2006.
